# Avocado Oil Prevents Kidney Injury and Normalizes Renal Vasodilation after Adrenergic Stimulation in Hypertensive Rats: Probable Role of Improvement in Mitochondrial Dysfunction and Oxidative Stress

**DOI:** 10.3390/life11111122

**Published:** 2021-10-21

**Authors:** Cristian Adrián Márquez-Ramírez, Berenice Eridani Olmos-Orizaba, Claudia Isabel García-Berumen, Elizabeth Calderón-Cortés, Rocío Montoya-Pérez, Alfredo Saavedra-Molina, Alain Raimundo Rodríguez-Orozco, Christian Cortés-Rojo

**Affiliations:** 1 Instituto de Investigaciones Químico—Biológicas, Universidad Michoacana de San Nicolás de Hidalgo, Morelia 58030, Mexico; qbio_cris@yahoo.com.mx (C.A.M.-R.); 1028309h@umich.mx (B.E.O.-O.); claudiaberumen07@live.com (C.I.G.-B.); rocio.montoya@umich.mx (R.M.-P.); francisco.saavedra@umich.mx (A.S.-M.); 2 Facultad de Enfermería, Universidad Michoacana de San Nicolás de Hidalgo, Morelia 58000, Mexico; elizabeth.calderon@umich.mx; 3 Facultad de Ciencias Médicas y Biológicas “Dr. Ignacio Chávez”, Universidad Michoacana de San Nicolás de Hidalgo, Morelia 58020, Mexico; alain.rodriguez@umich.mx

**Keywords:** hypertension, prazosin, adrenergic receptors, kidney disease, complex I, mitochondria, oxidative phosphorylation, renal vasculature, ROS, glutathione

## Abstract

Hypertension impairs the function of the kidney and its vasculature. Adrenergic activation is involved in these processes by promoting oxidative stress and mitochondrial dysfunction. Thus, the targeting of mitochondrial function and mitochondrial oxidative stress may be an approach to alleviate hypertensive kidney damage. Avocado oil, a source of oleic acid and antioxidants, improves mitochondrial dysfunction, decreases mitochondrial oxidative stress, and enhances vascular function in hypertensive rats. However, whether avocado oil improves the function of renal vasculature during the adrenergic stimulation, and if this is related to improvement in renal damage and enhancement of mitochondrial activity is unknown. Thus, the effects of avocado oil on renal vascular responses to adrenergic stimulation, mitochondrial dysfunction, oxidative stress, and renal damage were compared with prazosin, an antagonist of α_1_-adrenoceptors, in hypertensive rats induced by L-NAME. Avocado oil or prazosin decreased blood pressure, improved endothelium—dependent renal vasodilation, prevented mitochondrial dysfunction and kidney damage in hypertensive rats. However, avocado oil, but not prazosin, decreased mitochondrial ROS generation and improved the redox state of mitochondrial glutathione. These results suggest that avocado oil and prazosin prevented hypertensive renal damage due to the improvement in mitochondrial function.

## 1. Introduction

Hypertension is a health issue whose increasing incidence and prevalence worldwide has urged the search for novel approaches for its treatment [1]. Excessive production of reactive oxygen species (ROS) is one of the main factors involved in the pathogenesis and the development of the complications of this disease [2,3,4,5]. Therefore, the targeting of excessive ROS production is a potential candidate to treat this disease and its complications.

Exacerbated responses to several vasoconstrictor agents contribute to hypertension development, leading to an imbalance between sympathetic vasoconstriction and nitric oxide (NO^•^)—dependent vasodilation [6]. This imbalance is largely due to the combined activity of α_1_-adrenergic receptors and the AT_1_ receptors for angiotensin II [7]. The activation of these receptors increases the production of ROS via protein kinase C (PKC) and NADPH oxidase (Nox) [8,9,10,11], causing oxidative stress and damage to biomolecules and cell structures. The mitochondrial electron transport chain (ETC) is one of the targets of damage by Nox—derived ROS. The oxidative damage in the ETC leads to an increment in mitochondrial ROS production. Mitochondrial ROS activates endothelial Nox, enabling a feedback loop that perpetuates mitochondrial damage, ROS production, and Nox activation. This drives to a sustained decrease in NO**^•^** bioavailability and decreased endothelium—dependent vasodilation. The blocking of this vicious circle with mitochondria—targeted antioxidants decrease blood pressure in murine models of hypertension, evidencing the central role of mitochondria in the pathogenesis of hypertension [8].

Vascular dysfunction plays a central role in renal hypertensive damage by exacerbating vascular contraction and activating signaling pathways involved in the development of fibrosis and kidney damage. In this regard, the targeting of vascular oxidative stress generated by mitochondria can delay kidney damage in hypertensive animals [12]. We have previously reported that dietary supplementation with avocado oil, a source of a variety of bioactive molecules with antioxidant properties, improves the redox state of mitochondria in different organs during diabetes [13,14,15]. Moreover, avocado oil decreased blood pressure in hypertensive rats and improves kidney vascular function during the contraction induced by the stimulation of the AT_1_ receptors [16]. Whether avocado oil counteracts enhanced renal vasoconstriction induced by adrenergic stimulation, and if this is related to improvement in renal damage and enhancement of mitochondrial activity is unknown. Therefore, we tested the effects of avocado oil intake on renal vasoconstriction induced by adrenergic stimulation, kidney histology, mitochondrial function, and oxidative stress in L-NAME—induced hypertensive rats.

## 2. Materials and Methods

### 2.1. Animals and Experimental Groups

Forty male Wistar rats weighing 250–350 g were randomly distributed in five groups of eight animals each: control (CTRL) group; control plus avocado oil (CTRL+AO) group; hypertensive (HT) group; hypertensive plus avocado oil (HT+AO) group; hypertensive plus prazosin (HT+PZS) group. All the experimental groups were fed ad libitum with a standard rodent diet (Laboratory Rodent Diet 5001, LabDiet, St. Louis, MO, USA) and water. To induce hypertension in the HT, HT+AO, and HT+PZS groups, 75 mg/kg b.w. of L-NAME was added to the drinking water for one week. Subsequently, the L-NAME dose was reduced to 50 mg/kg of L-NAME for the next 45 days.

Avocado oil was supplemented daily by gavage at a dose of 3.6 g/kg b.w. for 45 days, parallel to the administration of 50 mg/kg of L-NAME. Water was administered by gavage instead of avocado oil in the CTRL group for the same time as avocado oil. Prazosin was given to the HT+PZS group at a dose of 1 mg/kg b.w. for 45 days, parallel to the administration of 50 mg/kg of L-NAME. 

A commercial presentation of avocado oil (Ahuacatlán, DIRICOM S.A. de C.V., Ciudad de México, México) was purchased from a local grocery. Avocado oil was from the same batch of the oil used in [14], whose fatty acid content consisted of 55.9% C18:1, 24.9% C18:2, 10.1% C18:3, 5.92% C16:0, 1.86% C18:0, 0.2% C16:1, and 0.92% of an unidentified fatty acid [14]. Prazosin hydrochloride, an α1-adrenoceptor selective antagonist, was from FARMASIERRA S.A. (San Sebastián de los Reyes, Madrid, Spain). L-NAME, a blocker of the nitric oxide synthase, was purchased from Sigma-Aldrich (St. Louis, MO, USA).

All the procedures with animals were carried out according to the recommendations issued by the Mexican Ministry of Agriculture in the paragraph of Federal Regulations for the Use of Animals (NOM-062-ZOO-1999). This research was also approved by the institutional committee for the use of animals of the Instituto de Investigaciones Químico Biológicas, Universidad Michoacana de San Nicolás de Hidalgo.

### 2.2. Blood Pressure Measurement

Blood pressure records were obtained by plethysmography with a CODA monitor (Kent Scientific, Torrington, CT, USA). The rats were placed in a holder five min before the determinations. Three systolic and diastolic blood pressure recordings were carried out each day for each animal during the last three days of treatment. All the procedures were performed according to the manufacturer’s instructions.

### 2.3. Renal Histology

After the sacrifice of the animals, both kidneys were resected and fixed in 10% paraformaldehyde, embedded in paraffin blocks, and sectioned at 3 µm for staining with hematoxylin-eosin. Stained glass slides were examined by light microscopy with a Leica DM5500B Microscope (Leica Microsystems, Wetzlar, Germany) by a researcher blinded to the experimental groups, who searched for changes in the architecture of the glomeruli, the Bowman’s capsule, and inflammation in the distal and proximal convoluted tubules.

### 2.4. Analysis of Renal Vascular Reactivity

Renal vascular function was assessed at the end of the treatments with concentration-response curves to agents inducing vasoconstriction or vasodilation. The rats were anesthetized with sodium pentobarbital at an intraperitoneal dose of 55 mg/kg. The right renal artery was cannulated, the kidney was resected and placed in a Langendorff system. The kidney was perfused with Krebs-Henseleit buffer (118 mM NaCl, 20 mM NaHCO_3_, 11.7 mM glucose, 4.7 mM KCl, 2.5 mM CaCl_2_, 1.2 mM KH_2_PO_4_, 1.2 mM MgSO_4,_ and 0.026 mM EDTA, pH 7.4) at 37 °C. The buffer was bubbled with a mixture of 95% O_2_ and 5% CO_2_, the liquid flow rate was adjusted to 10 mL/min. Before the start of the experimental protocol, the baseline perfusion pressure was allowed to stabilize for one hour.

Renal vasoconstrictor responses were evaluated by measuring the changes in perfusion pressure that caused the addition of 10 µL of phenylephrine at μM concentrations of 0.1, 0.31, 1, 3.1, 10, 31, 100, 310, 1000, 3100, and 10,000. Changes in perfusion pressure were monitored with a Grass FT03 type pressure transducer (Astro-Med, Inc., West Warwick, RI, USA). Renal vascular relaxation was evaluated in separate experiments with carbachol ((2-hydroxyethyl)trimethylammonium chloride carbamate), a parasympathomimetic drug that induces endothelium-dependent vasodilation. After the kidneys were perfused and stabilized as mentioned above, an infusion of 100 μM of phenylephrine was administered at a constant rate of 10 mL/h to induce vasoconstriction. Once the perfusion pressure stabilized, concentration-response curves to carbachol were made by adding 10 µL of carbachol at μM concentrations of 1, 10, 100, 1000, 10,000, and 100,000.

### 2.5. Mitochondria Isolation

Rats were sacrificed by decapitation at the end of the treatments and kidney mitochondria were isolated by differential centrifugation according to the protocol described elsewhere [15]. Mitochondrial protein concentration was determined by a modification of the Biuret method [17].

### 2.6. Analysis of ROS Levels and Mitochondrial Glutathione

ROS production was determined in kidney mitochondria by measuring the oxidation of the fluorescent probe 2′7′-dichlorodihydrofluorescein diacetate (H_2_DCF-DA). 0.5 mg/mL mitochondria and 1.25 mM of H_2_DCFDA were incubated at 4 °C for 20 min under constant stirring in a buffer containing 10 mM HEPES, 100 mM KCl, 3 mM MgCl_2,_ and 3 mM KH_2_PO_4_ (pH 7.4). Then, the mitochondrial suspension was placed in a quartz cell and the analysis started by recording the baseline fluorescence. 1 min later, 10 mM glutamate/malate was added to fuel the ETC in complex I, and the changes in the fluorescence were monitored for 20 min. Fluorescence was measured with a Shimadsu RF-5301PC spectrofluorophotometer (λ_ex_ = 485 nm; λ_em_ = 520 nm). The rate of ROS production was expressed as the changes in fluorescence in arbitrary units (a.u.) per minute, which was calculated by subtracting the fluorescence obtained 20 min after the addition of the substrate minus the fluorescence before the addition of the substrate and divided by twenty (ΔF/min). The levels of glutathione and its redox status were assessed in isolated mitochondria by a modification of the method by Akerboom and Sies [18], as reported elsewhere [15].

### 2.7. Assessment of Mitochondrial Function

Freshly isolated kidney mitochondria were resuspended in a final volume of 2 mL buffer containing 10 mM HEPES, 100 mM KCl, 3 mM MgCl_2_, and 3 mM KH_2_PO_4_ (pH 7.4). The mitochondrial suspension was placed in a sealed glass chamber with constant stirring. The oxygen consumption rate was determined at 37 °C using a Clark-type oxygen electrode coupled to a YSI 5300A oxygen monitor and a computer for data acquisition. Determinations started with the addition of 10 mM glutamate/malate as a respiratory substrate for complex I, or 10 mM succinate for complex II, and oxygen uptake was monitored for five min. Then, 1 mM ADP was added to stimulate the phosphorylating state (state 3). 1 mM oligomycin was added five min later to inhibit ATP synthase and stimulate respiration in state 4. The respiration rate was calculated from the slopes of the traces of oxygen consumption according to the instructions of the manufacturer of the oxygen monitor. The respiratory control ratio (RCR) was calculated by dividing the rate of respiration in state 3 by the rate of respiration in state 4.

### 2.8. Data Analysis

All data were expressed as the mean ± standard error. The number of individual experiments (n) is indicated in the legend of each figure. The statistical significance of the data was determined by one-way analysis of variance (ANOVA), followed by Tukey *post-hoc* test, except by Figure 4, where Student’s *t*-test was used. Analyses were carried out with the Statistica software (StatSoft, Hamburg, Germany)

## 3. Results

### 3.1. Effects of Avocado Oil or Prazosin on Blood Pressure

**Figure 1:**
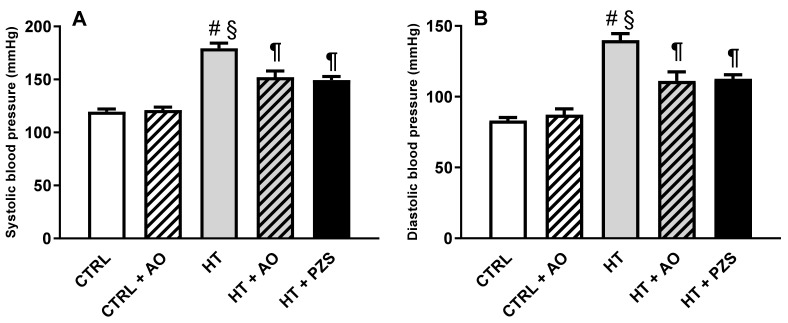
Effects of avocado oil (AO) or prazosin (PZS) on systolic (**A**) and diastolic (**B**) blood pressure of hypertensive rats (HT). Blood pressure was measured three days before the end of the treatments. The data are expressed as the mean ± s.e. of *n* ≥ 6. Statistical significances: ^#^ *p* > 0.05 vs. CTRL; ^§^ *p* < 0.05 vs. CTRL+AO; ^¶^ *p* < 0.05 vs. HT (one-way ANOVA, followed by Tukey’s *post hoc* test).

Systolic and diastolic blood pressures increased in the HT group (Figure 1A,B), respectively. Avocado oil decreased systolic and diastolic blood pressures in the HT+AO group without reaching the levels of the CTRL group. A similar effect was observed with prazosin in the HT+PZS group. Avocado oil did not have any effect, neither in systolic nor diastolic blood pressures in the CTRL+AVO group. These results indicate that avocado oil has an antihypertensive effect similar to that of prazosin, without affecting blood pressure in normotensive rats.

### 3.2. Effects of Avocado Oil or Prazosin on Renal Vascular Function in Hypertensive Rats

**Figure 2:**
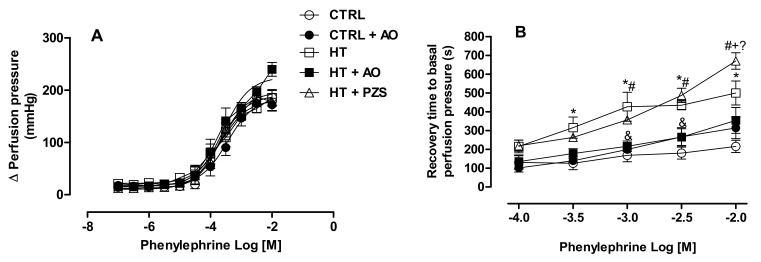
Effects of avocado oil (AO) or prazosin (PZS) on renal adrenergic vasoconstriction (**A**) and recovery time to basal perfusion pressure after adrenergic vasoconstriction (**B**). The data are expressed as the mean ± s.e. of *n* ≥ 6. Statistical significances: * CTRL vs. HT; ^#^ CTRL vs. HT+PZS; ^&^ HT vs. HT+AO; ^+^ HT vs. HT+PZS; ^?^ HT+AO vs. HT+PZS (one-way ANOVA, followed by Tukey’s *post hoc* test [*p* < 0.05]).

The effects of prazosin or avocado oil on renal vasoconstriction induced by α-adrenergic stimulation were addressed in perfused kidneys (Figure 2A). No differences in the vasoconstriction stimulated by phenylephrine were observed in any experimental group with respect to the CTRL group. The time of the return of renal vasculature to basal perfusion pressure after the adrenergic stimulation was plotted in Figure 2B. The renal vasculature of the CTRL group backs to basal perfusion pressure earlier than the vasculature of the HT group at any concentration of phenylephrine. Avocado oil decreased the time of return to basal perfusion pressure in the HT+AO group in a statistically significant way at all the concentrations of phenylephrine, except at 0.01 M (i.e., the logarithmic molar concentration of −2 in the graph). In contrast, prazosin did not improve this parameter in the HT+PZS group; indeed, prazosin delayed the return to basal perfusion pressure above the time displayed by the HT group at 0.01 M phenylephrine. 

**Figure 3:**
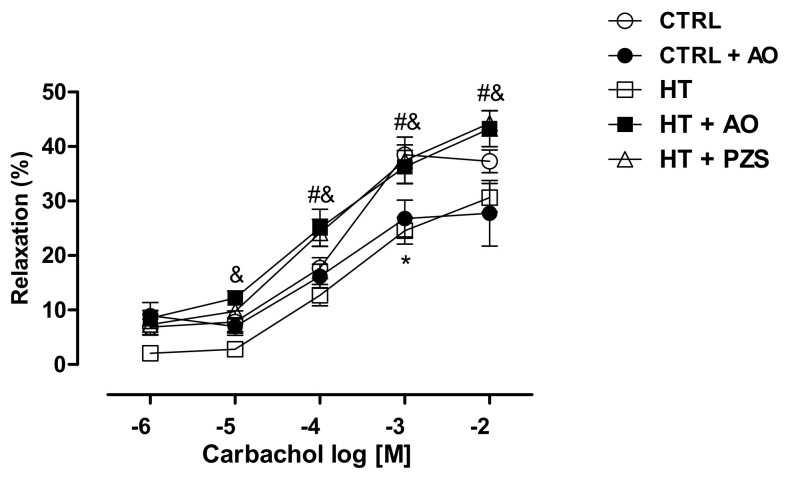
Effect of avocado oil (AO) or prazosin (PZS) on renal endothelium-dependent vasodilation after the vasoconstriction induced by phenylephrine. The data are expressed as the mean ± s.e. of *n* ≥ 4. Statistical significances: * CTRL vs. HT; ^#^ HT vs. HT+PZS; ^&^ HT vs. HT+AO (One-way ANOVA, followed by Tukey’s *post hoc* test. [*p* < 0.05]).

The effects of avocado oil or prazosin on endothelium-dependent vasodilation after the vasoconstriction induced by phenylephrine are shown in Figure 3. The kidney vasculature of the HT group displayed a decreased response to carbachol, a muscarinic agonist, in comparison to the CTRL group (Figure 3). In contrast, both avocado oil and prazosin fully counteracted impaired cholinergic vasodilation in the HT+AO and HT+PZS groups, respectively.

**Figure 4:**
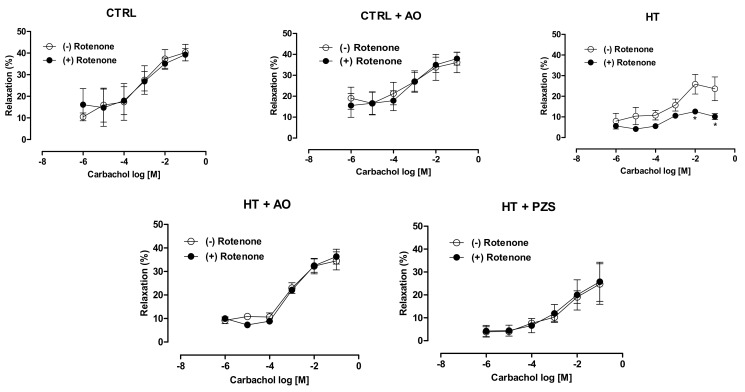
Effects of rotenone on renal muscarinic vasodilation after the vasoconstriction induced by phenylephrine in rats treated with avocado oil (AO) or prazosin (PZS). The data are expressed as the mean ± s.e. *n* ≥ 4. * *p* < 0.05 vs. HT group without rotenone (-) (Student’s *t*-test).

Endothelium-dependent vasodilation was assessed in the presence of rotenone, an inhibitor of mitochondrial complex I, to assess whether avocado oil or prazosin may counteract impaired renal vasodilation by mitochondrial ROS. As shown in Figure 4, vasodilation was severely impaired in the kidneys of the HT group. Avocado oil or prazosin prevented this effect in the kidneys of the HT+AO and HT+PZS groups, respectively. Rotenone did not alter the vasodilation in the kidneys of the CTRL and CTRL+AO groups. Overall, these results indicate that avocado oil and prazosin prevent the impairment in endothelium-dependent vasodilation after the vasoconstriction elicited by adrenergic stimulation in hypertensive rats. This may be related to the decrease of mitochondrial oxidative stress, as suggested by the protective effect of avocado oil or prazosin on impaired vasodilation induced by rotenone in hypertensive rats.

### 3.3. Effects of Avocado Oil and Prazosin on Mitochondrial Oxidative Stress

**Figure 5:**
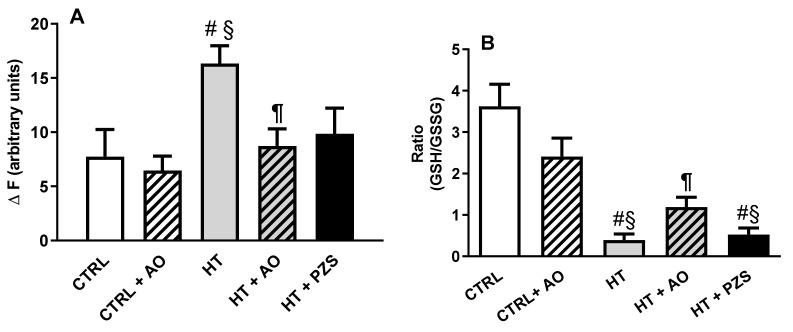
Effects of avocado oil (AO) or prazosin (PZS) on mitochondrial oxidative stress in kidney of hypertensive (HT) rats. (**A**) ROS levels in isolated mitochondria fueled in the complex I of the electron transport chain with glutamate/malate. (**B**) Reduced-to-oxidized glutathione ratio (GSH/GSSG) in isolated kidney mitochondria. The data are expressed as the mean ± s.e. of *n* ≥ 3. Statistical significances: ^#^ *p* > 0.05 vs. CTRL; ^§^ *p* < 0.05 vs. CTRL+AO; ^¶^ *p* < 0.05 vs. HT (One-way ANOVA, followed by Tukey’s *post hoc* test).

ROS levels in kidney mitochondria fueled in the complex I with glutamate/malate are shown in Figure 5A. ROS levels in the HT group doubled the levels in the CTRL group. Avocado oil completely prevented this effect, as observed in the HT+AO group, while prazosin just tends to decrease ROS levels, as observed in the HT+ PZS group. The redox state of glutathione in kidney mitochondria was determined by assessing the ratio of reduced-to-oxidized glutathione (GSH/GSSG) (Figure 5B). The GSH/GSSG ratio was nine times lower in the HT group than in the CTRL group. Avocado oil increased threefold the GSH/GSSG ratio in the HT+AO group with respect to the HT group, while prazosin did not have any effect on this parameter, as observed in the HT+PZS group. In summary, only avocado oil significantly improves the oxidative stress induced by hypertension in kidney mitochondria.

### 3.4. Effects of Avocado Oil and Prazosin on Mitochondrial Function 

**Figure 6:**
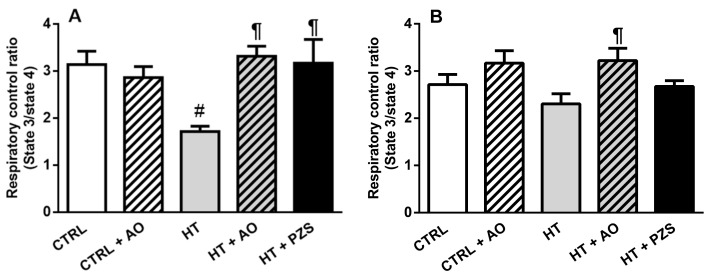
Effects of avocado oil (AO) or prazosin (PZS) on respiratory control ratio (RCR) of kidney mitochondria from hypertensive (HT) rats, treated with avocado oil (AO) or prazosin (PZS). Mitochondrial respiration was fueled in the complex I with glutamate/malate (**A**) or in the complex II with succinate (**B**). The data are expressed as the mean ± s.e. of *n* ≥ 4. Statistical significances: ^#^ ***p*** > 0.05 vs. CTRL; ^¶^ *p* < 0.05 vs. HT (One-way ANOVA, followed by Tukey’s *post hoc* test).

Respiratory control ratio (RCR), a parameter that reflects the efficiency of the mitochondrial oxidative phosphorylation, is shown in Figure 6. The RCR decreased by 45.2% in the HT group when mitochondria were fueled with a complex I substrate (Figure 6A). This was fully prevented by avocado oil or prazosin in the hypertensive rats, as observed in the HT+AVO and HT+PZS groups, respectively. Avocado oil did not affect the RCR in normotensive rats, as observed in the CTRL+AVO group. On the other hand, RCR was affected neither in the HT group nor in any of the remaining experimental groups when succinate, a complex II substrate (Figure 6B), fueled mitochondrial respiration. These results indicate that hypertension caused mitochondrial dysfunction in the complex I of the mitochondrial electron transport chain, which was counteracted by avocado oil or prazosin.

### 3.5. Effects of Avocado Oil or Prazosin on Renal Function of Hypertensive Rats

**Figure 7:**
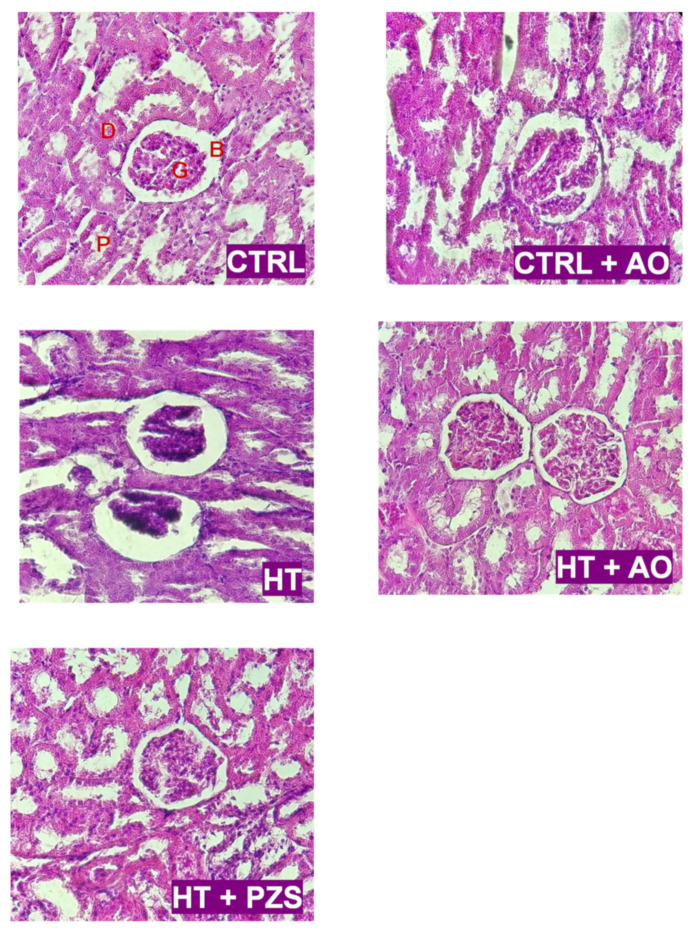
Effects of avocado oil (AO) or prazosin (PZS) on the renal architecture of hypertensive (HT) rats. Kidney sections were stained with hematoxylin & eosin and observed at 20×. B: Bowman’s capsule; D: Distal convoluted tubule; G: Glomerulus; P; Proximal convoluted tubule. Images are representative of four independent observations using different kidney samples.

The effects of avocado oil and prazosin on hypertensive kidney damage were evaluated by the staining of kidney slides with hematoxylin-eosin and examination by light microscopy (Figure 7). The kidney sections of the CTRL and CTRL+AO groups have a normal architecture with well-defined glomeruli (G) and Bowman’s capsule (B), while no inflammatory changes were detected in the distal (D) and proximal (P) convoluted tubules. The HT group displayed fragmented and empty glomeruli, an increment in the Bowman’s capsule area, and poor light in both the distal and proximal convoluted tubules. The changes in the architecture of both the glomeruli and convoluted tubules detected in the HT group were not observed in the HT+AO and HT+PZS groups. Moreover, the latter groups exhibited a decrease in the Bowman’s capsule area. These results indicate that avocado oil prevented hypertensive kidney damage to the same degree as prazosin, which may be mediated by the decrease in mitochondrial dysfunction induced by avocado oil and prazosin. 

## 4. Discussion

The results of this study show that avocado oil decreased systolic and diastolic blood pressure in hypertensive rats with the same efficacy that prazosin, an α1-adrenoceptor antagonist (Figure 1). This was accompanied by improvement of endothelium-dependent vasodilation of the renal vasculature (Figure 2B and Figure 3), and prevention of impaired renal vasodilation due to mitochondrial dysfunction by rotenone (Figure 4). Despite avocado oil and prazosin improving the mitochondrial function (Figure 6), only avocado oil decreased mitochondrial oxidative stress (Figure 5), although both treatments prevented kidney injury (Figure 7). The decrease in oxidative stress obtained with avocado oil may be related to that besides improving vasodilation after adrenergic stimulation (Figure 2B and Figure 3), avocado oil also enhances vasodilation after stimulation of AT_1_ receptors in hypertensive rats [16]. Thus, it can be inferred that avocado oil has a dual mechanism consisting in the improvement of endothelium—dependent vasodilation regardless renal blood vessels are constricted either by α_1_- adrenergic or AT_1_ receptor stimulation. 

Another factor by which avocado oil may decrease blood pressure and improve renal vasodilation is its high oleic acid content. It has been suspected that the low incidence of cardiovascular diseases in Mediterranean populations is due in part to the high consumption of olive oil, another source of oleic acid [19,20]. On the other hand, the α_1_- adrenergic receptors are G-protein coupled receptors, whose activity is dependent on the fatty acid composition of membranes, being oleic acid an inhibitor of G-proteins [21]. Accordingly, it has been shown that oleic acid has a hypotensive effect by modulating the activity of G-proteins, leading to an increment in vasodilatory stimuli [19]. Thus, it can be suggested that the antihypertensive effect of avocado oil is due to the modulation by oleic acid of the G-proteins mediating the vasoconstriction stimulated by the activation of adrenergic receptors, which is exacerbated in hypertension [6].

As mentioned above, it has been shown that the decrease in mitochondrial oxidative stress in the endothelium diminishes systemic blood pressure in rats with hypertension [8]. This antihypertensive effect has been attributed to the increase in the bioavailability of NO^•^ due to lower peroxynitrite formation (ONOO^−^) because of lower superoxide production in the mitochondrial ETC. This seems to be in conflict with the low levels of NO^•^ observed in the blood of hypertensive rats treated with avocado oil [16] and the diminution in the levels of mitochondrial oxidative stress observed in Figure 5. However, it must be pointed out that hypertension was induced in this study by the chronic administration of L-NAME, a competitive inhibitor of the nitric oxide synthase (NOS). Thus, the increment in NO^•^ levels by avocado oil should not be expected, despite decreased mitochondrial oxidative stress in the HT+AVO group. 

Rotenone induces ROS production in the complex I of the ETC [22]. Figure 4 shows that rotenone severely impaired the endothelium-dependent vasodilation of renal vasculature in hypertensive rats, which further supports the idea that excessive mitochondrial ROS production is involved in the impaired vascular function in hypertension. Moreover, this result also agrees with a report about the role of ROS production in the complex I in the development of hypertension [23]. The negative effect of rotenone on vasodilation was not observed in the HT+AVO and HT+PZS groups, indicating that these treatments prevented the production of ROS in the complex I that impaired vasodilation in the HT group. This is in concordance with the lower ROS production observed in the HT+AVO group and the non-statistically significant tendency to lower ROS production in the HT+PZS group (Figure 5A). Increased antioxidant capacity induced by avocado oil has been previously observed by our group in mitochondria from the liver, kidney, and brain of diabetic rats [13,14,15,24]. This could be due to the presence of β-sitosterol in avocado oil [25], which modulates the activity of antioxidant enzymes in mitochondria [26] and acts as an antioxidant and stabilizer in membranes [27]. In addition, various carotenoids in avocado oil also have antioxidant effects [28]. 

It can be argued that rotenone decreases ROS production when driven by the reverse electron transfer (RET) from succinate to NAD^+^, as it has been observed in isolated mitochondria fueled with succinate as the only respiratory substrate [29]. Several observations argued against the physiological occurrence of this mode of ROS production and its inhibition by rotenone [30] and references therein. (i) The concentrations of succinate used to induce RET-ROS (2.5–5 mM) are way above the physiological range of succinate concentrations (0.1–1 mM). Indeed, negligible ROS generation by RET is observed with physiological concentrations of succinate [30]. (ii) There is a very unfavorable thermodynamic barrier for electron transfer from ubiquinol to NAD^+^, and the conditions used to overcome this barrier (high concentrations of succinate and NAD^+^, low concentrations of fumarate and NADH, and the presence of complex III and complex IV inhibitors), are not of physiological relevance [30]. (iii) It has been shown that in vivo exposure to rotenone increases ROS production due to its interaction with the complex I [31,32]. Therefore, ROS production by RET and its inhibition by rotenone is highly unlikely in the model of perfused kidneys, as it does not involve either the exposure to high concentrations of succinate and NAD^+^ or the incubation with complex III or complex IV blockers. Moreover, the increased ROS production in the perfused kidney by rotenone may be favored by the mitochondrial dysfunction (Figure 6A) and the higher ROS production with a complex I substrate (Figure 5A) observed in kidney mitochondria of the HT group.

On the other hand, the non-statistically significant trend of prazosin to decrease ROS might be related to that the antagonism of α1-adrenergic receptors inhibits the diacylglycerol-protein kinase C (DAG-PKC) pathway. This pathway is involved in ROS production by activating Nox in endothelial cells, vascular smooth muscle cells, and renal mesangial cells [33]. Therefore, the antagonism of α1-adrenergic receptors by prazosin may mediate a decrease in ROS production by Nox, leading to improvement in mitochondrial function, as observed in the HT+PZS group (Figure 6A). However, it must be stressed that prazosin did not improve the antioxidant capacity of mitochondria from hypertensive rats, as reflected by the lack of positive effects in the GSH/GSSG ratio of the HT+PZS group (Figure 5B). This was expected since to our knowledge, prazosin is not an antioxidant molecule per se and does not stimulate the activity of antioxidant systems. In contrast, avocado oil did decrease oxidative stress in the HT+AVO group (Figure 5B), which is consistent with the presence in avocado oil of a variety of antioxidant molecules that increase antioxidant capacity in mitochondria as stated above.

The diminution of the RCR in the HT group only with a substrate of the complex I indicates that hypertension inhibits the activity of the complex I. Complex I dysfunction is an important source of ROS and oxidative stress in various pathological conditions [34,35]. Oxidative stress has been proposed as a main cause of complex I inhibition via the oxidation of reactive protein thiols. This depends on the mitochondrial GSH/GSSG ratio: the lower GSH/GSSG ratio, the higher inhibition of the complex I [36]. On this basis, it can be suggested that hypertension induces oxidative stress in renal mitochondria by the over-stimulation of the adrenergic system [37], leading to over-activation of the DAG-PKC pathway and enhanced ROS production by vascular Nox [10,11]. As proposed by Dikalova et al., [8], Nox-derived ROS would lead to mitochondrial oxidative stress, causing exhaustion of antioxidant defenses, as reflected by the decrease in the GSH/GSSG ratio (Figure 5B). In turn, this would drive glutathionylation of the complex I [36], causing impairment in oxidative phosphorylation when mitochondrial ETC is fueled with a complex I substrate (Figure 6A). The antagonism of α_1_-adrenergic receptors may block excessive Nox activation and damage to complex I function. This is supported by the protective effect of prazosin against the decrease in the RCR with glutamate-malate (Figure 6A). However, this would not be enough to counteract other pathways contributing to mitochondrial oxidative stress in hypertension, such as the over-activation of AT_1_ receptors [38], explaining why the GSH/GSSG ratio remained low in the HT+PZS group. In contrast, the presence in avocado oil of antioxidants like β-sitosterol would drive enhanced GSH/GSSG ratio in the group HTZ+AVO (Figure 5B), preventing the inhibition of oxidative phosphorylation fueled with a complex I substrate in this group (Figure 6A) and decreasing ROS levels (Figure 5A). Besides, avocado oil may be protecting from oxidative stress produced by other systems in hypertension, such as the stimulation of AT_1_-receptors, as shown previously by our group [16].

Different murine models of hypertension show histological signs of glomerular damage. This was proposed to be a consequence of mitochondrial alterations, including impairment in respiration and ATP synthesis, and increment in oxidative stress and ROS levels [12,39]. This is in line with our results in the HT group of glomerular damage (Figure 7), impaired oxidative phosphorylation (Figure 6A), augmented ROS levels (Figure 5A), and diminished GSH/GSSG ratio (Figure 5B). Moreover, it has been shown that the decrease in blood pressure without amelioration of mitochondrial dysfunction and oxidative stress does not prevent the development of hypertensive kidney disease [12]. Accordingly, it was observed that avocado oil or prazosin prevented glomerular damage in the HT+AO and HT+PZS groups, respectively (Figure 7). This may be the consequence that avocado oil decreased mitochondrial ROS generation, increased the GSH/GSSG ratio (Figure 5), and enhanced oxidative phosphorylation (Figure 6A). In contrast, prazosin improved oxidative phosphorylation (Figure 6A), but neither decreased ROS levels nor augmented the GSH/GSSG ratio (Figure 5). This suggests that the improvement in mitochondrial function with prazosin may be enough to prevent hypertensive kidney damage, independently of a decrease in mitochondrial oxidative stress.

It is tempting to assume that large amounts of avocado oil should be consumed by humans to achieve the benefits observed in rats. This happens if the dose of avocado oil used in rats is converted to an equivalent dose in humans based only on body weight. We have calculated the equivalent dose of avocado oil that a human should consume by the body surface area (BSA) normalization method [40]. Considering a density of avocado oil of 0.8942 g/mL, and the dose of 3.5768 × 10^4^ mg/kg used in this study, it turns out that the human equivalent dose is 579.79 mg/kg. That means than an individual weighing 70 kg should consume 47.7 mL of avocado oil per day. This dose is similar to that recommended for olive oil in humans, where the daily consumption of four tablespoons (i.e., ~60 mL) has been associated with lower incidence of cardiovascular events [41]. It must be stressed that we are not advocating the usage of this avocado oil dose, as the safety of this or other avocado oil dose remained to be investigated in humans.

## 5. Conclusions

Avocado oil decreased systemic blood pressure and improved endothelium-dependent vasodilation in the kidneys of hypertensive rats subjected to adrenergic stimulation. This was accompanied by kidney damage alleviation, which may be a consequence of the improvement in mitochondrial function and oxidative stress. Comparatively, the antagonism of α_1_-adrenergic receptors with prazosin prevented hypertensive kidney damage, improved mitochondrial function, enhanced endothelium-dependent vasodilation after the adrenergic stimulation of kidney vasculature, and decreased systemic blood pressure in hypertensive rats. Nevertheless, prazosin did not improve mitochondrial oxidative stress, suggesting that prevention of hypertensive kidney damage by prazosin may be independent of a decrease in mitochondrial oxidative stress. Overall, the results of this study suggest that avocado oil may have the potential like a nutritional adjuvant to counteract the negative effects of hypertension on the kidney.

## Data Availability

The raw data supporting the conclusions of this article will be made available by the corresponding author, without undue reservation.
